# The Rate and Risk Factors of Postpartum Depression in Vietnam From 2010 to 2020: A Literature Review

**DOI:** 10.3389/fpsyg.2021.731306

**Published:** 2021-10-27

**Authors:** Huong Thi Thanh Nguyen, Anh Phuong Hoang, Ly Thi Kim Do, Stephen Schiffer, Huyen Thi Hoa Nguyen

**Affiliations:** ^1^College of Health Sciences, Vin University, Hanoi, Vietnam; ^2^Faculty of Nursing and Midwifery, Hanoi Medical University, Hanoi, Vietnam; ^3^47B General Surgery Department, University Medical Center Schleswig-Holstein, Lübeck, Germany

**Keywords:** literature review, prevalence, risk factors, Vietnam, postpartum, depression

## Abstract

**Background:** Postpartum depression (PPD) is commonly seen in women after birth and can lead to adverse effects on both the health of mothers and child(ren) development. In Vietnam, there have been a number of studies examining the rate and risk factors of PPD, but none has provided a systematic review.

**Aim:** This current literature review aims to summarize and synthesize the current state of knowledge of studies conducted in Vietnam to provide a comprehensive understanding of the PPD phenomena during the last 10 years.

**Data Sources:** A literature search was conducted relying on the most common online databases—MEDLINE/PubMed, ScienceDirect, and Google Scholar, which included articles if they (i) examined prevalence or risk factors of PPD; (ii) were conducted among Vietnamese participants using either quantitative, qualitative, or mixed-method, and (iii) were published from 2010 to 2020. After the filtering process, 18 articles were eligible to be reviewed.

**Results:** Research studies in Vietnam on PPD are conducted among women at and after 1-month delivery. The rate of PPD reported in Vietnam among mothers at postnatal time points from 1 to 12 months ranged from 8.2 to 48.1%. Risk factors can be clustered into three groups: personal factors, family factors, and environmental factors.

**Recommendation:** Further research studies should focus on examining PPD at an earlier stage within the first month after birth. The investigation of risk factors in a comprehensive manner for Vietnamese mothers would also be recommended.

## Background

Globally, postpartum depression (PPD) is a significant mother health issue in the first year after giving birth. According to the 10th revision of International Classification of Diseases (ICD-10), PPD in maternity patients aged 12–55 years is defined by the ICD code F53.0 (World Health Organization, 2010). This can be considered as either mood disorder or mental illness, which is characterized by restless, anxious, fatigued, and worthless, and depressed mood, low energy, and even suicidal ideation commonplace (Stewart et al., 2003). Depression with its onset at week 4–6 may last for more than 2 weeks or up to 1 year or later with some cases needing professional care (Stewart et al., 2003). Additionally, previous studies indicated other common mental health changes after birth. Baby blues (transient emotional lability phase) and postpartum adjustment, which both start within early weeks postpartum, are mostly recovered without treatment (Helle et al., 2016; Mokhtaryan et al., 2016). Postpartum anxiety (including both clinical psychiatric disorders and a dimensional level of general anxiety) and postpartum psychosis (very rarely) occurring within the first 2 weeks, but the duration can be weeks or months and almost all of cases required hospitalization due to its severity (Stewart et al., 2003; Stewart and Vigod, 2016). However, a large number of recently published articles with findings varying across countries focus only on the prevalence of PPD as the most common mental disorder. Among Asian regions, the prevalence was 36% in Pakistan (Husain et al., 2006), 30.2% in Taiwan (Chien et al., 2006), or 16.8% in Thailand (Limlomwongse and Liabsuetrakul, 2006).

Risk factors associated with PPD include personal issues such as a low level of education background (Do et al., 2018; Wesselhoeft et al., 2020) or a history of mental health disorders (Silverman et al., 2017; Tho Tran et al., 2018). Other reasons, such as infant gender among areas where local residents remained traditionally unscientific belief (Murray et al., 2015), intimate partner violence (Tho Nhi et al., 2019), or stressful life events (SLEs) like a job loss, death of loved one, or an economic shock (Qobadi et al., 2016; Gausman et al., 2020), can worsen the situation of PPD. Prospective studies have shown that PPD may also lead to impaired physical, mental development for children and lower life quality, or even suicidal or self-harming behaviors of a mother (Herba et al., 2016; Gressier et al., 2017; Haddad et al., 2017; Tungchama et al., 2017). In Vietnam, there have been a number of studies about the rate and risk factors of PPD using different screening, including the Edinburgh Postnatal Depression Scale (EPDS) (Chen et al., 2012; Van Vo et al., 2017; Do et al., 2018; Wesselhoeft et al., 2020), Self-Reporting Questionaire-20 (SRQ-20) (Upadhyay et al., 2019; Gausman et al., 2020), or diagnostic guide as the Diagnostic and Statistical Manual of Mental Disorders (DSM) (Nguyen et al., 2015). This current literature review aims to summarize and synthesize the current state of knowledge about PPD in Vietnam. The central question is what is the PPD rate and the risk factors examined among Vietnamese women?

## Method

### Search Strategies

**Figure 1:**
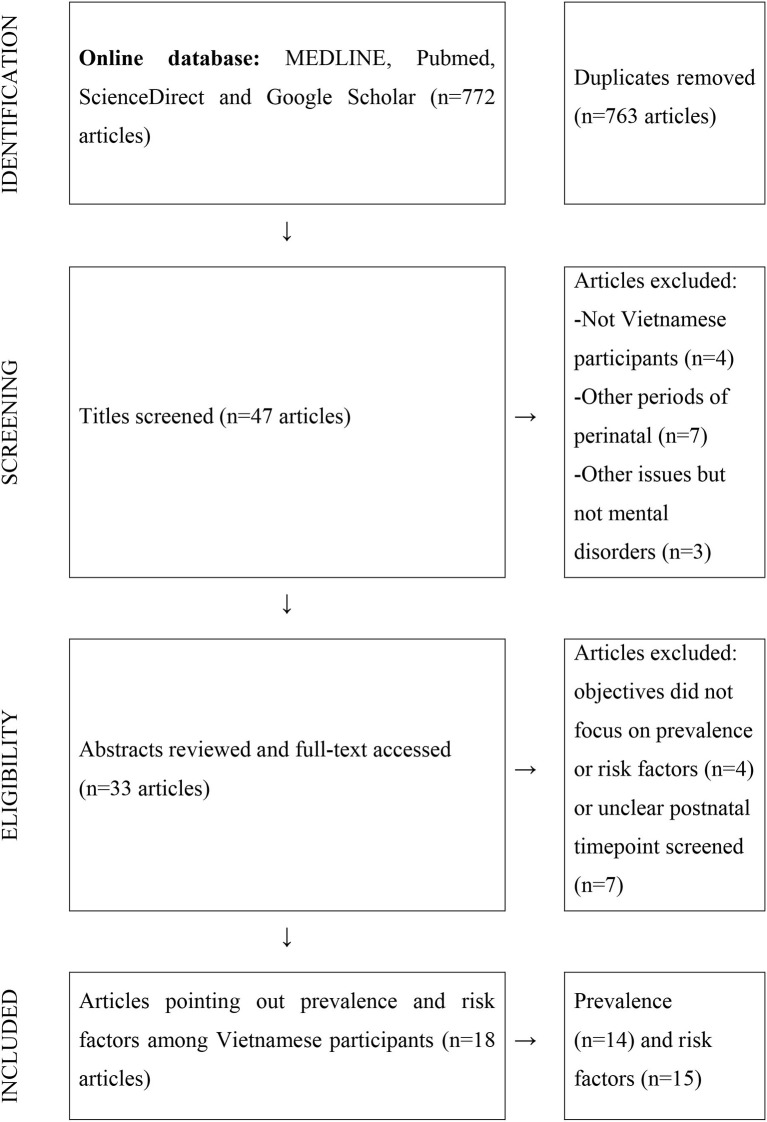
PRISMA Flow diagram (Liao et al., 2019).

A literature search was conducted on the following online databases: MEDLINE/PubMed, ScienceDirect, and Google Scholar. In both Vietnamese and English, search terms included Vietnam, depressive^*^, postpartum were used separately and as combination during the search. Only research articles published within the last 10 years matching these key terms were eligible. The reference list of published articles led to other related studies. Following the initial search, all results, titles, abstracts, and full texts were filtered and reviewed before being included in this study. Some studies that examined both prevalence and risk factors were only counted as one. Figure 1 illustrates the search process.

### Inclusion and Exclusion Criteria

Articles were included if they (i) examined the prevalence of risk factors of postpartum common mental disorders with any measurement scale; (ii) were conducted among Vietnamese participants using either quantitative, qualitative, or mixed methods. While quantitative studies illustrated obvious numbers and possible associations between PPD and its related predictors, qualitative research presented experiences of new mothers in the contexts of their socio-cultural; and (iii) were published within the last 10 years from 2010 to 2020.

### Analysis Method

Eligible articles were reviewed by two separate individuals in order to ensure the credibility of the findings. A literature review matrix as below (Table 1), which included author, year, objectives, setting, measurement tool, time point screened, prevalence, risk factors, was used to criticize articles.

**Table 1:** Literature matrix.

**No**	**Title**	**References**	**Objectives**	**Participants**	**Design**	**Sample size**	**Settings**	**Tools**	**Postnatal time point screened**	**Prevalence**	**Risk factors**
1	Common perinatal mental disorders in northern Viet Nam: community prevalence and health care use	Fisher et al. (2010)	To establish the prevalence of common perinatal mental disorders, and their association with preventive health care used among women in one rural and one urban province in northern Viet Nam	Women at least 7 months pregnantor between the fourth and eighth week postpartum	A cross-sectional quantitative study	364	Hanoi and Hanam	DSM diagnosis criteria	4–8 weeks	29.9%	Living in rural provinces, exposed to intimate partner violence, fearful of other family members, exposed to coincidental life adversity.
2	Adversity, social capital, and mental distress among mothers of small children: A cross-sectional study in three low and middle-income countries	Gausman et al. (2020)	To examine the relationship between adversity, cognitive social capital and mental distress among mothers of young children in three low and middle-income countries.	Mothers between 6- and 18-months post-partum	A quantitative study	5,485 women (n = 1,703 in Ethiopia, n = 11,792 in India, and n = 1,849 in Vietnam)	Ethiopia, India, and Vietnam	20-Question Self-Reporting Scale (SRQ-20). Cutting point for VN: 8 points	6–18 months	21% in Vietnam	Stressful life events
3	Association between unintended births and risk of postpartum depression: Evidence from Ethiopia, India, Peru and Vietnam	Upadhyay et al. (2019)	To examine the association between birth intention and the risk of PPD	Those who had delivered their babies in 5 to 21 months prior to the survey	Part of cohort study	1,811 women in Ethiopia, 1,800 women in India, 1,992 women in Peru and 1,835 women in Vietnam	Ethiopia, India, Vietnam, Peru	Self-reported-questionnaire (SRQ-20)	5–21 months	24% in Vietnam suffered from PPD	
4	Associations of Psychosocial Factors with Maternal Confidence among Japanese and Vietnamese Mothers	Goto et al. (2010)	To investigate the prevalence and associated sociodemographic, parenting, and psychological characteristics of low maternal confidence in child rearing among them	Mothers who had children 1-3m had	A cross-sectional study	294 Vietnamese women	Tu Du Obstetrical and Gynecological Hospital	GSE scale and a two-question case-finding instrument (Whooley et al., 1997)	1–3 months	23%	Young age, being a first-time mother and having achieved higher educational levels were significantly more frequent in the group lacking confidence.
5	Mothers' feeling for the first time of delivery	Tran (2010)	To explore the emotions of first-time mothers and factors that influence mothers' emotions to make recommendations to help mothers and family members better understand the complex emotions of mothers in certain childcare and parenting situations.	Mothers (20 to 38 years old) with children between 1 and 2 years old.	A mix of qualitative and quantitative study, questionnaire survey, opinion poll, in-depth interview	Interview: 10 mothers; survey: 60 mothers	Hoan Kiem District, Thanh Tri District, and Thanh Xuan District	In-depth interview using open-ended questions.	Over 1 year and 2 years		Mental preparation when the baby is born Husband's sharing concern Physiological characteristics of the child Mother's knowledge and experience of raising children The unity of husband and wife, mother-in-law in raising children.
6	Common mental disorders among women, social circumstances and toddler growth in rural Vietnam: a population-based prospective study	Fisher et al. (2015)	To examine the effect of maternal common mental disorders (CMD) and social adversity in the post-partum year on toddler's length-for-age index in a rural low-income setting.	Baseline: Women in late pregnancy or 4–6 weeks post-partum.Followed up (15 months later): the women and their toddlers.	A population-based prospective cohort study	211	6/116 communes in Ha Nam randomly selected	Diagnostic and Statistical Manual of Mental Disorders	Baseline: Women in late pregnancy or 4–6 weeks post-partum. Followed up (15 months later): the women and their toddlers.	33.6% (pregnancy or 4-6w postnatal)18.5% (15 months)	Interpersonal violence in her own childhood, a relationship with the intimate partner that is characterized by coercion, control, and a lack of affection and kindness, and lack of dedicated care during the first 30 post-partum days, all occurring in the context of poverty.
7	Emotional violence exerted by intimate partners and postnatal depressive symptoms among women in Vietnam: A prospective cohort study	Tran et al. (2018)	To investigate the association between various types of emotional experience during life with present partner and postnatal depressive symptoms among women in Vietnam.	Pregnant women	Cohort study	1,274 pregnant women	24 communes in the Dong Anh District, Hanoi, Vietnam.	Edinburgh Postpartum Depression Scale (EPDS)	4-12 weeks Interviewed 4 times: (a) at enrolment (before week 24 of pregnancy); (b) at a gestational age of 30–34 weeks; (c) 24–48 hours after delivery; and (d) 4–12 weeks after delivery.	8.2%	Emotional violence (physical, sexual, and verbal) as risk factors for postnatal depressive symptoms.
8	Intimate Partner Violence among Pregnant Women and Postpartum Depression in Vietnam: a Longitudinal Study	Tho Nhi et al. (2019)	To investigate the association between exposure to emotional violence, physical violence, and sexual violence during pregnancy and postpartum depression among women in northern Vietnam	The first one was conducted when participants were at least at 24 weeks' gestation and the second when participants were at a gestational age of 30–34 weeks. After childbirth two assessments of mothers were done at delivery and 4–12 weeks after delivery.	A longitudinal study	1274 women	Dong Anh Hospital and Bac Thang Long Hospital in Dong Anh district, Hanoi, Vietnam	EPDS/ cutoff 10	4–12 weeks after delivery OR at delivery	8.2%	Both physical and sexual violence were statistically significantly associated with postpartum depression.
9	The relationship between cultural factors, mental trauma and risk of postpartum depression among mothers in Thuong Tin, Hanoi.	Le (2015)	To examine the current mental health problems of mothers before giving birth (6–9 months) and after giving birth (3 months). To understand some socio-cultural factors and psychological traumas that are risk factors of PPD in mothers.	6–9 months pregnant mothers and 3 months postpartum	A qualitative study	Phase 1: 144 answer sheets, phase 2: 134 answer sheets that meet the selection criteria.	Thuong Tin. Hanoi, Vietnam	EPDS (cutoff 10).	3 months postnatal and 6–9 months pregnant	14.9%	Conflicts with the husband's family, lack of support from friends and colleagues, the child's gender expectations are not as expected.
10	Perinatal common mental disorders among women and the social and emotional development of their infants in rural Vietnam	Tran et al. (2014)	To examine the effect of exposures to maternal symptoms of ante- and post-natal CMD on infant social–emotional development in a low-income setting.	Women from pregnant time until 6 months postpartum (before 20 gestational weeks (Wave 1, W1), at about 28 weeks gestation (Wave 2, W2), and, with their infants, at 6 weeks (Wave 3, W3) and 6 months (Wave 4, W4) postpartum.)	Prospective community-based investigation and follow-up study	378	Ha Nam province	EPDS-V, cut-off 4 points	8 weeks and 6 months postpartum (PPD)	At 8 weeks: 10,8%At 6 months: 12,4%	
11	Postnatal depressive symptoms amongst women in Central Vietnam: a cross-sectional study investigating prevalence and associations with social, cultural and infant factors	Murray et al. (2015)	(1.) To estimate the prevalence of postnatal depressive symptoms amongst women in Central Vietnam and (2.) To explore the influence of social, cultural and infant factors on the postnatal emotional wellbeing of women in a culturally distinct province of Vietnam.	Mothers from selected communes	A cross-sectional survey	465	Six urban and six rural communes in Thua Thien Hue	EPDS cut-off of 12/13	1-6 months	37.1 % for possible depression	Level of education. Confinement practices. Infant gender was not significant correlated with depression The amount a child cried, breastfeeding difficulties, and diarrhea in the past two weeks The reactions of family members to infant - Social support - Being classed as poor, food insecurity, experiencing violence in the past 12 months - Being frightened of family members, being frightened of your husband
12	Postpartum change in common mental disorders among rural Vietnamese women: incidence, recovery and risk and protective factors	Nguyen et al. (2015)	To determine the incidence and rates of recovery from common mental disorders (CMD) among rural Vietnamese women and the risk and protective factors associated with these outcomes from the perinatal period to 15 months after giving birth.	Mothers in the last 3 months of pregnancy or the first 4–6 weeks postpartum; follow up until 15 months later	A population-based prospective study	211	Ha Nam province	DSM-IV. assessed by psychiatrist administered Structured Clinical Interview for Disorders	1 year	18,5%	Having a sustained period of mandated rest and heightened postpartum care before having to resume usual responsibilities, not having experienced intimate partner violence in the prior year and having practical support with household work and infant care The experience of childhood maltreatment and intimate partner providing little care, sensitivity, kindness or affection and the chronic stress of household poverty.
13	Postpartum depression and parental self-efficacy: a comparison of native Korean and Vietnamese immigrant mothers in Korea	Choi et al. (2012)	To compare postpartum depression and parental self-efficacy between married immigrant women from Vietnam and native Korean mothers	Mothers within 12 weeks of giving birth.	A quantitative study	141 women (72 native Korean mothers and 69 immigrantVietnamese mothers)	A suburban city in South Korea	EPDS (cutoff 13).	12 weeks	34.3%	
14	Postpartum Depressive Symptoms and Associated Factors in Married Women: A Cross-sectional Study in Danang City, Vietnam	Van Vo et al. (2017)	To (1) estimate the prevalence of PPD symptoms among married women in one Vietnam city (Danang) and (2) identify the social and personal factors associated with postpartum depressive symptoms.	Women who gave birth 4 weeks to 6 months prior to being interviewed.	A cross-sectional study	600	Hai Chau District, Danang, Vietnam	EPDS (cutoff point of 12/13).	4-week to 6-month postnatal.	19.3%	Not being able to rely on their husband for help, having a husband who does not spend time to discuss problems, having anxiety about matters other than the birth, not exercising after giving birth, having an ill baby.
15	Symptom Endorsement and Sociodemographic Correlates of Postnatal Distress in Three Low Income Countries	Nguyen et al. (2016)	To (1) compare endorsement of specific symptoms by mothers meeting criteria for maternal distress in these three settings and (2) evaluate the consistency of associations between maternal distress and recognized risk factors.	5647 mothers in Ethiopia, India (Andhra Pradesh), and Vietnam participating in an ongoing cohort study (Young Lives)	A cross-sectional, secondary analysis	5,647 (Vietnam = 1,855)	Ethiopia, Andhra Pradesh, and Vietnam	SRQ-20.The Self-Reporting Questionnaire-20 Items (SRQ-20),	6–18 months	21.2%	Negative life events and the index child experiencing either a life-threatening event or long-term health problem. Living in an urban setting (OR = 2.82), experiencing an economic shock (OR = 2.34); participating in any livelihood activities.
16	Emotional violence and maternal mental health: a qualitative study among women in northern Vietnam	Tran et al. (2018)	To explore Vietnamese women's experiences of emotional partner violence and their perceptions of the implications of such violence for their mental health.	10 pregnant and 10 recently given birth	A qualitative study	20 women of the 1,337 pregnant women who reported exposure to emotional partner violence and attained high depression scores in prospective cohort	Antenatal care facilities in Ðông Anh district	Edinburgh postnatal depression scale (EPDS). Cutting point/scale 30 points (10 items)	4 times: at enrolment (at a gestational age of less than 24 weeks); at a gestational age of 30–34 weeks; 24–48 h after delivery; and 4–12 weeks after delivery		Emotional violence: being ignored; being denied support; and being exposed to controlling behaviors
17	Perceptions and experiences of perinatal mental disorders in rural, predominantly ethnic minority communities in northern Vietnam	Abrams et al. (2016)	To investigate knowledge/ experiences and perceptions of perinatal mental disorders (PMDs) and their treatments at the community level in a rural, predominantly ethnic minority region of northern Vietnam.	Primary health workers (PHWs) working at local community health centers, and pregnant or postpartum women enrolled in a program for maternal and infant health	Qualitative semi-structured interviews.	14 perinatal women and 12 PHWs	Four communes located within the Dinh Hoa district of Thai Nguyen province	Two vignette scenarios, one based on DSM-IV	Either pregnant or women in their first-year postpartum		Family relationships impact psychological well-being Both traditional and western medicine play roles in perinatal health. Lack of personal knowledge of women experiencing PMDs.
18	Postnatal depressive symptoms display marked similarities across continents	Wesselhoeft et al. (2020)	To examine and compare the factor structure of postnatal depressive symptoms measured by EPDS in postpartum women from Denmark, Vietnam and Tanzania.	Women who were part of one of the three pregnancy cohorts: Denmark- early pregnancy and up until 2.5 months postpartum; Vietnam and Tanzania: early pregnancy and up until gestational age 24 weeks inVietnam and 30 weeks in Tanzania	A cross-sectional study	4,516 (Vietnam: 1,278)	Denmark, Vietnam and Tanzania.	EPDS cut off point of 12 and above	40–90 days postpartum		The highest level of education (level 3) was associated with a significantly lower EPDS total score, when adjusting for country (*p* < 0.001)

## Results

### Characteristics of Articles About Postpartum Depression in Vietnam Published From 2010 to 2020

The search strategies resulted in 47 abstracts from studies conducted among Vietnamese participants. Among those, 14 articles were excluded due to unrelated objectives that did not examine any rate or risk factors. Next, 15 articles were eliminated after a full-text review, which indicated that the postnatal screening time point was unclear or focused on antenatal or perinatal period instead of postpartum. In the end, 18 research articles met the criteria to be reviewed in this literature review.

As presented in Table 2 regarding the characteristics of eligible articles, from 2010, it can be seen that four studies (22.22%) used a qualitative approach, whereas others were undertaken using quantitative methods, including a cross-sectional study (*n* = 8; 44.45%) or a cohort study (*n* = 6; 33.33%). About 83% (*n* = 15) studies had a sample size of more than 100 mothers. The research settings included four (22.22%) studies conducted at commune health centers, five (27.78%) at district hospitals, five (27.78%) at provincial hospitals, and four (22.22%) at national hospitals. Tools and guidelines used to screen or diagnose PPD differed from each study. The most frequently used screening tool was the EPDS regardless of different versions or cutoff point (*n* = 9; 50%), followed by DSM diagnosis criteria (*n* = 4; 22.22%), SRQ-20 (*n* = 3; 16.67%), and others (*n* = 2; 11.11%). Postnatal screening time for PPD was between 1 and 3 months (*n* = 8; 44.45%), between 4 and 6 months (*n* = 4; 22.22%), and up to or later than 12 months (*n* = 6; 33.33%).

**Table 2:** Characteristics of articles about postpartum depression in Vietnam published from 2010 to 2020.

**Characteristics**		**Frequency (*N* = 18)**	**Percentage(%)**
Research settings where studies were conducted	Commune level	4	22.22
	District level	5	27.78
	Province/City level	5	27.78
	Country level	4	22.22
Design of studies	Cross-sectional study	8	44.45
	Cohort study	6	33.33
	Qualitative study	4	22.22
Sample size(Quantitative research only)	<100 participants	3	16.67
	100–1000 participants	9	50
	>1,000 participants	6	33.33
Measurement scale of PPD	EPDS	9	50
	DSM diagnosis criteria	4	22.22
	SRQ-20	3	16.67
	Other^*^	2	11.11
Postnatal time points screened	0–1 m	0	0
	1–3 m	8	44.45
	3–6 m	4	22.22
	5–12 m (>12 m)	6	33.33

* *Other measurement scales: a two-question case-finding instrument (Whooley et al., 1997) and self-designed questionnaire with Likert scale from 1–4 and 1–5*.

### Rate of Postpartum Depression in Vietnam Reported by Articles Published From 2010 to 2020

**Figure 2:**
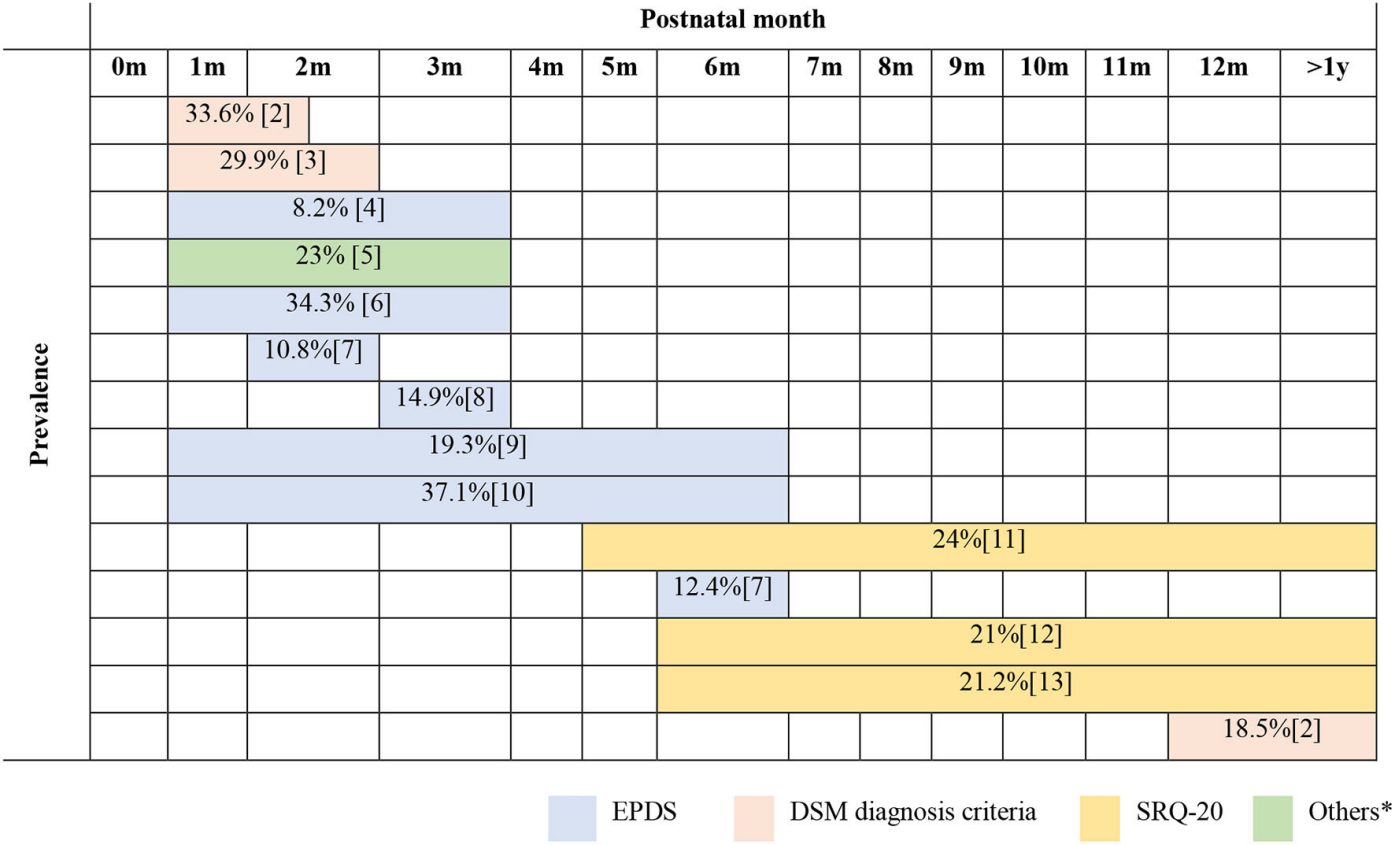
Prevalence of postpartum common mental disorders against postnatal screening time points reported by published articles in Vietnam from 2010 to 2020.

The time diagram (Figure 2) illustrated the rate of PPD following the postnatal screening time points from 1 month after birth. Among articles that met the criteria to be chosen for review, 14 (77.78%) of them examined the prevalence of PPD with a clearly defined time point. Most studies used the EPDS measurement scale (*n* = 9; 50%).

No study was performed among Vietnamese participants in the early time after birth, especially days within the first 2 weeks. From the first month to the third month, research revealed a wide range for the rate of PPD. The highest reported rate was ~34% (33.6 and 34.3%) in two different research settings. One study used EPDS to compare the rate of PPD between married, immigrant women from Vietnam and native Korean mothers within 12 weeks (Choi et al., 2012). The other used DSM diagnosis criteria to examine the effect of maternal common mental disorders and social adversities in the postpartum year in a rural low-income setting in Vietnam (Fisher et al., 2015). In contrast, the lowest rate of PPD (8.2%) was screened by EPDS in a part of a cohort study investigating the association between emotional experience with a present partner and postnatal depressive symptoms among women in Vietnam (Tho Tran et al., 2018).

Two studies performed between the first and the sixth month demonstrated a high rate of PPD. Both studies used EPDS with a similar sample size (465 and 600 participants) and research settings to estimate the rate of postnatal depressive symptoms amongst women in central Vietnam, ranging from 19.3 to 37.1% (Murray et al., 2015; Van Vo et al., 2017).

Four studies examined a later postnatal time of up to more than 1 year. In these studies, the rate was about 20% (18.5–24%). Three out of four studies looking at this period used the SRQ-20 measurement scale (Nguyen et al., 2016; Upadhyay et al., 2019; Gausman et al., 2020). These studies shared common features as design (a cross-sectional study), sample range (>1,000 participants), and research setting (at country level).

### Risk Factors of Postpartum Depression Reported by Articles in Vietnam Published From 2010 to 2020

As summarized in Table 3, there are a number of risk factors contributing to PPD, as revealed in 16 studies. Findings from both quantitative and qualitative research were included to illustrate the association between PPD and risk factors.

**Table 3:** Risk factors of PPD reported by articles in Vietnam from 2010 to 2020.

**Factors**		**Indicated by research in Vietnam**	
		**The number of qualitative studies revealed**	Strength of the relationship in quantitative studies^*^
Personal factors	Low level of education (Murray et al., 2015; Tho Tran et al., 2018; Wesselhoeft et al., 2020)	2	*OR* = 2.17; 95% CI: 1.21–3.89
			*p* < 0.001 (OR were not reported)
	Poor knowledge and lack of experience about PPD (Tran, 2010; Abrams et al., 2016)	2	
	Unwell-prepared psychology to be a mother (Tran, 2010; Tran et al., 2014)	2	
	Unsatisfaction about new life after birth (Le, 2015)	3	
	History of mental trauma (Fisher et al., 2015; Murray et al., 2015; Tho Tran et al., 2018)	1	*OR* = 4.12; 95% CI: 2.06–8.21
			*OR* = 2.44; 95% CI: 1.51–3.94
Family factors	Lack of family support (Fisher et al., 2010; Tran, 2010; Le, 2015; Nguyen et al., 2015; Van Vo et al., 2017; Tho Tran et al., 2018; Tho Nhi et al., 2019)	4	*OR* = 3.53; 95% CI: 2.19–5.70
			*OR* = 3.36; 95% CI: 1.05–10.71
			*OR* =3.46; 95% CI: 1.87–6.39
	Poor relationship with family members (Tran et al., 2014, 2018; Murray et al., 2015; Abrams et al., 2016)	4	
	Problems with child's health (Tran, 2010; Murray et al., 2015; Nguyen et al., 2016; Van Vo et al., 2017; Tho Tran et al., 2018)	3	*OR* = 2.20; 95% CI: 0.96–5.03
			*OR* = 1.60
	Suffered from intimate physical/ emotional/ sexual violence (Fisher et al., 2010; Tran et al., 2014; Tho Nhi et al., 2019)	1	*OR* = 2.11; 95% CI: 1.12–3.96
			*OR* = 5.08; 95% CI: 2.58–10.02
	Husband's preference of son (Tho Tran et al., 2018; Tho Nhi et al., 2019)	0	*OR* = 1.78; 95% CI: 1.01–3.13
			*OR* = 1.98; 95% CI: 1.15–3.39
Environmental factors	Stressful life events (an economic shock or coincidental life adversity) (Fisher et al., 2010; Tran et al., 2014; Le, 2015; Nguyen et al., 2016; Gausman et al., 2020)	3	*OR* = 4.40; 95% CI: 2.44–7.93
			*OR* = 2.34
	Rural living area (Nguyen et al., 2015, 2016)	2	*OR* = 2.82
	Lack of social support/reaction (Murray et al., 2015; Nguyen et al., 2016)	2	*OR* = 4.4
	Confinement practices (Murray et al., 2015; Nguyen et al., 2015)	2	

* *Each row represents the result of 1 study*.

Among personal factors, a low level of education was pointed out as a risk factor of PPD. In particular, mothers with primary or secondary school education had a higher risk of PPD compared to ones with high school education [odds ratio (OR) = 2.17; 95% CI: 1.21–3.89 and OR = 3.55; 95% CI: 1.74–7.25, respectively]) in a study by Tho Tran et al. (2018). Similarly, research by Wesselhoeft et al. in 2020 concluded that the highest level of education (university/college) was associated with a significantly lower EPDS total score when adjusting for country (*p* < 0.001) (Wesselhoeft et al., 2020). Poor knowledge, lack of experience about PPD, poorly prepared to be a mother from a psychological perspective, and feeling unsatisfied about new life after birth were revealed as personal factors leading to PPD in qualitative studies (Tran, 2010; Le, 2015; Abrams et al., 2016). Finally, new mothers with a history of mental trauma were more likely to be exposed to PPD, as indicated in both qualitative (Abrams et al., 2016) and quantitative studies (Tho Tran et al., 2018).

Family factors can be considered as a major cause of PPD. Lack of family support appeared to be a predictor of PPD in three quantitative studies with an OR of about 3.5 (Fisher et al., 2010; Tho Tran et al., 2018; Tho Nhi et al., 2019). Poor relationships with family members and lack of family support were also identified as risk factors of PPD in case of happening together in qualitative studies (Tran, 2010; Fisher et al., 2015; Murray et al., 2015; Tran et al., 2018). Mothers caring for a sick child were more likely to be predisposed to PPD, as indicated in studies with OR of 1.6 and 2.2, respectively (Nguyen et al., 2016; Tho Tran et al., 2018). Intimate partner violence, including physical, emotional, and sexual violence, was revealed as a risk factor of PPD in quantitative studies (OR ranged from 2.11 to 5.08) (Fisher et al., 2010; Tho Nhi et al., 2019). Lastly, the preference for a son by the husband was also a typical and cultural risk factor of PPD for his wife, with an OR between 1.78 and 1.98 (Tho Tran et al., 2018; Tho Nhi et al., 2019).

With regard to environmental factors, SLEs appeared to be a significant factor leading to PPD of new mothers. SLEs, such as an economic shock or coincidental life adversity, revealed a strong association with PPD as indicated in two quantitative studies (*OR* = 2.34; *OR* = 4.40, respectively) (Fisher et al., 2010; Nguyen et al., 2016). Living in rural areas and lack of social support/reaction were also found as risk factors of PPD in two qualitative studies (Abrams et al., 2016). These risk factors were also confirmed in quantitative studies that people living in a rural area and lack social support had 2.82- and 4.4-times higher risk of PPD exposure, respectively (Fisher et al., 2010; Nguyen et al., 2016). The confinement, which refers to traditional practice, especially in Eastern countries, includes enforced rest, lying over heat, not bathing for a particular period, and following specific recipes (Murray et al., 2015). These practices beginning immediately after birth and lasting for a culturally variable length were risk factors for PPD, as mentioned in qualitative interviews about postnatal depressive symptoms among women in central Vietnam (Murray et al., 2015).

## Discussion

Eighteen eligible articles published in the last 10 years were reviewed and summarized in this literature review. The results reflected the differences in study designs, including the characteristics of participants, the use of screening scales, and the postnatal screening time point for PPD. All these components led to a wide range in the rate of PPD among Vietnamese participants (8.2–37.1%).

The different postnatal time points when PPD was screened can lead to various rates, especially during the early period after birth. Many studies about postpartum emotional disorders among women during few days/weeks after birth were alarming regarding its high prevalence (44.3% in Hong Kong, 31.3% in Nigeria, and 58% in India) (Mokhtaryan et al., 2016) and its prediction of further PPD (Reck et al., 2009). Postpartum psychosis, a more serious condition, was also mentioned during this time, however with lower rate, from 0.89 to 2.6 in 1,000 women in a systematic review (VanderKruik et al., 2017). However, no data were reported explicitly about the rate of PPD within the first month after birth. There is a definitive lack of data in the literature about this mental health issue at the early stage of within the first month after birth among Vietnamese participants during the last 10 years.

In many countries, EPDS was the most commonly used instrument to screen PPD (Klainin and Arthur, 2009; Hegde et al., 2012; Özcan et al., 2017; VanderKruik et al., 2017). Research by Santos et al. (2007), when comparing the validity of EPDS and SRQ-20, confirmed the reliability of EPDS for screening PPD with sensitivity 82.7% (74.0–89.4%) at the best cutoff point ≥10 and specificity 65.3% (59.4–71.0%) (Santos et al., 2007). In this literature review in Vietnam, EPDS, DSM diagnosis criteria, and a two-question case-finding instrument by Whooley et al. in 1997 were all used to point out the rate of PPD during the first 3 months. In particular, studies examined the earlier postnatal time (6 months or less) preferred EPDS, whereas studies conducted at time points later than 6 months used SRQ-20. Even so, the purposes of using EPDS, SRQ-20, and DSM were different from each other, leading to a variety of reported rates. However, while EPDS and SRQ-20 are considered as screening tools for mental disorders (PPD) by healthcare workers (Santos et al., 2007), DSM is diagnostic criteria for doctors or specialists in a confirmed diagnosis (De Jesus Mari and Williams, 1986; Frances et al., 1995; Cox and Holden, 2003).

Although the same measurement scale was used, the rate can still be different due to the variance in characteristics of participants. Most research in Vietnam focused on examining the PPD at the postnatal time points of between 1 month and 3 months, with the rate from 8.2 to 34.3%. Regardless of using EPDS, while research by Tho Nhi et al. in 2019 among women at 4 and 12 weeks after delivery in 24 communities in Dong Anh District (Hanoi, Vietnam) pointed to a lower rate at 8.2% (Tran et al., 2018), a study conducted in Korea to compare PPD between married immigrant women from Vietnam and native Korean mothers revealed a much higher rate at 34.3% (Choi et al., 2012). This difference came from characteristics of participants and research settings. Participants from the study conducted in Korea were immigrant women vulnerable to depression due to more SLEs or cultural shock (O'Mahony et al., 2013). Conversely, research by Tho Nhi et al. in 2019 revealed results similar to the prevalence of a cross-sectional study conducted in Portugal with mothers whose children aged from 15 days to 3 months (11.8%) and a study in Japan examined mothers at the age-specific 3 months after birth (14.8%) (Matsumoto et al., 2011; Silva et al., 2017).

The differences in the reported rate of PPD may be caused by how PPD was defined, how PPD scales were translated, or the cutting point of PPD scales. The assessment of PPD at the postnatal time point of 6 months in this literature review showed significant differences in the rate of PPD (19.3 and 37.1%) despite similar sample sizes and research settings. About 37.1% was the rate at risk of PPD, with the cutting point of EPDS scale at 9/10 (Murray et al., 2015), whereas 19.3% was the rate for probable depression with the cutting point of EPDS at 12/13 (Van Vo et al., 2017). A study conducted in Korea to measure depression and/or anxiety among women 6 months after delivery using the EPDS scale with the cutting point of 12/13 also revealed a prevalence of 14.3% (Yeo, 2006). In comparison to some Asian countries with the similar culture, including India, Thailand, and Indonesia (the prevalence is 11–15, 8.4, and 18.37%, respectively), the prevalence of PPD in Vietnam is lower, whereas this prevalence is reported higher in a Taiwanese study (42.6%) (Chen et al., 2007; Hegde et al., 2012; Panyayong et al., 2013; Nurbaeti et al., 2018).

This literature review showed that studies conducted in more socio-economically developed areas revealed a lower rate of PPD at the period of 6 months after birth in Vietnam. It can be inferred that research settings may contribute as a risk factor of PPD. Research settings can be physical, social, and cultural sites (Given, 2008), including income, level of education, and information access. For instance, rural residents with economic issues or insufficiency of both mental health infrastructure and specialists are less likely to define themselves as needing care and lack of access to specialty mental health services (Gamm et al., 2010).

In terms of personal risk factors of PPD illustrated in research conducted in Vietnam, key points to consider can be lack of knowledge of mothers, lack of experience about PPD, and dissatisfaction about transitioning from previous life circumstances to a new reality. Providing essential information for new mothers was confirmed as a helpful method to reduce the risk of PPD in a study by Fiala et al. (2017). Based on the Knowledge, attitude and practices (KAP) model of Launiala in 2009, without sufficient knowledge, a mother cannot have the right attitude and proper practice regarding PPD prevention. Lack of preparation to be a mother and a history of mental trauma were mentioned as links to psychosocial factors in some research globally (Klainin and Arthur, 2009; Özcan et al., 2017).

Poor quality of relationships with family members and lack of family support were prominent causes of PPD. Research in India and Thailand reported marital conflict in depressed women as a factor independently associated with PPD (Hegde et al., 2012; Panyayong et al., 2013). A number of findings in other countries, such as Korea (Lee and Park, 2018), China (Xie et al., 2010), and the United States of America (Negron et al., 2013), emphasized the importance of support from family members, especially husbands to women with PPD. Xie et al. (2010) in a research related to Chinese culture also indicated that women might be more vulnerable to family support after birth due to physiological and psychological changes (Xie et al., 2010).

Most studies in Vietnam shared similar findings with those in India and Turkey that environmental factors, such as SLEs, were predictors of PPD (Hegde et al., 2012; Özcan et al., 2017; Upadhyay et al., 2019). A SLE can be the death of a close relative, assault, serious marital problems, or divorce/breakup, which was studied with an onset of an episode of major depression in women (Kendler et al., 2010). Additionally, cultural aspects, such as traditional confinement practice after birth or the preference for a son by the husband should be further assessed in relation to the PPD in Vietnam, following findings confirmed in Asian countries (Klainin and Arthur, 2009).

## Conclusion

Most research about PPD conducted in Vietnam among women in the first 3 or 6 months after delivery revealed a rate of PPD from 8.2 to 37.1%. Measurement tools, postnatal time points, and research settings can impact the rate of PPD. Risk factors may result from the own characteristics of mothers, family relationships, or social environment.

Further studies of PPD in Vietnam should focus on women within the first month after birth. Although symptoms and signs of PPD can appear within the first few days after delivery, currently no research in Vietnam has been conducted at this early postnatal time point. The EPDS is recommended to screen for PPD as its validity and reliability have been confirmed. A comprehensive questionnaire to examine risk factors of PPD for Vietnamese women should also be developed.

## Author Contributions

All authors listed have made a substantial, direct and intellectual contribution to the work, and approved it for publication.

## Conflict of Interest

The authors declare that the research was conducted in the absence of any commercial or financial relationships that could be construed as a potential conflict of interest.

## Publisher's Note

All claims expressed in this article are solely those of the authors and do not necessarily represent those of their affiliated organizations, or those of the publisher, the editors and the reviewers. Any product that may be evaluated in this article, or claim that may be made by its manufacturer, is not guaranteed or endorsed by the publisher.
